# Genomic and metabonomic methods reveal the probiotic functions of swine-derived *Ligilactobacillus salivarius*

**DOI:** 10.1186/s12866-023-02993-9

**Published:** 2023-08-30

**Authors:** Jiajun Yang, Peng Shang, Bo Zhang, Jing Wang, Zhenyu Du, Shanfeng Wang, Jun Xing, Hao Zhang

**Affiliations:** 1 School of Animal Husbandry and Veterinary Medicine, Jiangsu Vocational College of Agriculture and Forestry, Jurong, 212400 Jiangsu China; 2 Beijing Key Laboratory for Animal Genetic Improvement, College of Animal Science and Technology, China Agricultural University, Beijing, 100193 China; 3 College of Animal Science, Tibet Agriculture and Animal Husbandry College, Linzhi, 860000 China

**Keywords:** *Ligilactobacillus salivarius*, Probiotic, Metabonomic analysis, Metagenomic analysis

## Abstract

**Background:**

As substitutes for antibiotics, probiotic bacteria protect against digestive infections caused by pathogenic bacteria. *Ligilactobacillus salivarius* is a species of native *lactobacillus* found in both humans and animals. Herein, a swine-derived *Ligilactobacillus salivarius* was isolated and shown to colonize the ileal mucous membrane, thereby promoting nutritional digestion, absorption, and immunity. To evaluate its probiotic role, the entire genome was sequenced, the genetic information was annotated, and the metabolic information was analyzed.

**Results:**

The phylogenetic relationship indicated that the bacteria was closer to *L. salivarius* MT573555.1 and MT585431.1. Functional genes included transporters, membrane proteins, enzymes, heavy metal resistance proteins, and putative proteins; metabolism-related genes were the most abundant. The six types of metabolic pathways secreted by *L. salivarius* were mainly composed of secretory transmembrane proteins and peptides. The secretory proteins of *L. salivarius* were digestive enzymes, functional proteins that regulate apoptosis, antibodies, and hormones. Non-targeted metabolomic analysis of *L. salivarius* metabolites suggested that ceramide, pyrrolidone- 5- carboxylic acid, N2-acetyl-L-ornithine, 2-ethyl-2-hydroxybutyric acid, N-lactoyl-phenylalanine, and 12 others were involved in antioxidation, repair of the cellular membrane, anticonvulsant, hypnosis, and appetite inhibition. Metabolites of clavaminic acid, antibiotic X14889C, and five other types of bacteriocins were identified, namely phenyllactic acid, janthitrem G, 13-demethyl tacrolimus, medinoside E, and tertonasin. The adherence and antioxidation of *L. salivarius* were also predicted. No virulence genes were found.

**Conclusion:**

The main probiotic properties of *L. salivarius* were identified using genomic, metabonomic, and biochemical assays, which are beneficial for porcine feeding. Our results provided deeper insights into the probiotic effects of *L. salivarius*.

**Supplementary Information:**

The online version contains supplementary material available at 10.1186/s12866-023-02993-9.

## Introduction

*Lactobacillus* is an important genus of probiotic bacteria found in some organs of humans and animals [1, 2]. Some *Lactobacillus sp.* species are used as probiotics owing to their myriad of benefits [3, 4], such as improvement of the gastrointestinal tract microbiome, production of the lactic acid and small peptides, defense against pathogens, and improvement in immunity [5–7]. Certain species of *Lactobacillus* sp. such as *Ligilactobacillus salivarius*, *Lactobacillus reuteri*, *Lactobacillus acidophilus*, *Lactobacillus bulgaricus*, and *Lactobacillus casei*, are regarded as safe by the Food and Drug Administration [8, 9]. Indeed, diets supplemented with these species can improve immunity and enhance growth through a reciprocal reaction with the host [10].

*Ligilactobacillus salivarius* (*L. salivarius*), a core bacterium in the gut, is a probiotic species used as a dietary supplement [11] since it can regulate stress, protect against pathogens, improve microbiological composition, and enhance growth [12–14]. We have previously shown that supplementation with *L. salivarius* improved body growth, reduced the rate of diarrhea through targeted colonization of the ileal mucous membrane, and improved the bacterial composition of weaning piglets [7, 15]. *L. salivarius* supplementation increases the relative abundances of beneficial bacteria of the genera *Lactobacillus*, *Acinetobacter*, *Ruminococcaceae* UCG-014, *Bacteroides*, and *Helicobacter* and decreases the abundance of strains of *Escherichia*-*Shigella*, *Streptococcus*, *Peptoclostridium*, *Roseburia*, and *Veillonella*. Metabolites secreted by *L. salivarius* can also promote the growth of beneficial bacteria and inhibit the growth of pathogens [7].

Genomic and metabonomic technologies have been widely used for functional gene annotation and identifying unknown metabolites [16–18]. In the present study, *L. salivarius* was sequenced using pan-genome and non-targeted metabonomic techniques to explore the functional genes owned and substances that were metabolized. In addition, virulence genes, other metabolites, interactions with the host, and drug-resistance genes were analyzed to determine the characteristics of the bacteria. The aim of this study is to determine the characteristics of *L. salivarius* isolated from healthy local Wei pig breeds to elucidate the potential mechanisms underlying its beneficial effects on the cecal microbiota, body antioxidant status, colonization of the ileum, and immune function in piglets. These findings will help facilitate future studies on the mechanism of the interplay between probiotics and hosts.

## Materials and methods

### Preparation of *L. salivarius*

*L. salivarius* was isolated from the ileum of healthy local Wei pigs as previously described [7]. All animal experiments were approved by the experimental guidelines of the Institutional Animal Care and Use Committee of China and the Institution of Animal Science and Welfare of Anhui Province, China (No. IASWAP2014100849). *L. salivarius* was stored in China General Microbiological Culture Collection Center (CGMCC), Beijing, China (storing number CGMCC17718). *L. salivarius* was cultured in de Man, Rogosa, and Sharpe medium (MRS) after inoculation at 1% in 37 ℃ for 16 h. Bacterial cells were harvested after approximately 18 h of fermentation. Deoxyribonucleic acid (DNA) samples were isolated from the cell pellets using a bacterial genome extraction kit (Sangon, SK8255, China) according to the manufacturer’s instructions, and quality control was subsequently carried out on the purified deoxyribonucleic acid (DNA) samples. The 16S ribosomal deoxyribonucleic acid (rDNA) was amplified and employed with polymerase chain reaction (PCR); the product was recycled and sequenced; 22 strains of sequences of *L. salivarius* were collected from the database of nucleotide in NCBI (https://www.ncbi.nlm.nih.gov/nuccore). Phylogenetic tree of *L. salivarius* was constructed and employed with MEGA 9.0 software and compared with the 22 strains from different origins (Supplementary Dataset) downloaded from the gene bank of NCBI.

### DNA extract and Illumina HiSeq sequencing

Genomic DNA was isolated from cell pellets using a Bacteria DNA Kit (OMEGA, D3350-00, GA, USA) according to the manufacturer’s instructions, and quality control was subsequently performed on the purified DNA samples. The genomic DNA was quantified using a TBS-380 fluorometer (Turner BioSystems Inc., Sunnyvale, CA, USA). A high-quality DNA sample (OD260/280 = 1.8–2.0, > 6 μg) was utilized to construct a fragment library.

For Illumina paired-end sequencing of bacteria, at least 3 μg of genomic DNA was used for sequencing library construction. Paired-end libraries with insert sizes of ~ 400 bp were prepared according to Illumina’s standard genomic DNA library preparation procedures. Purified genomic DNA was sheared into smaller fragments of the desired size using Covaris g-TUBE (Woburn, MA, USA), and blunt ends were generated using T4 DNA polymerase. After adding an ‘A’ base to the 3' end of the blunt phosphorylated DNA fragments, adapters were ligated to the ends of the DNA fragments. The desired fragments were purified via gel-electrophoresis, selectively enriched, and amplified using PCR. The index tag was introduced into the adapter at the PCR stage, as appropriate, and a library quality test was performed. Finally, a qualified Illumina paired-end library was used for Illumina HiSeq sequencing (PE150 mode, Shanghai BIOZERON Co., Ltd).

### Pacific Biosciences sequencing

For sequencing, the whole genome was broken into 20 kb segments, and 20 kb insert whole genome shotgun libraries were generated and sequenced using a Pacific Biosciences RS instrument. An aliquot of 8 μg DNA was centrifuged in a Covaris g-TUBE (Covaris, MA) at 6,000 rpm using an Eppendorf 5, 424 centrifuge (Eppendorf, NY, USA). DNA fragments were then purified, end-repaired, and ligated with SMRTbell sequencing adapters following the manufacturer’s instructions (Pacific Biosciences, CA, USA). The resulting sequencing libraries were purified three times using 0.45xvolumes of Agencourt AMPure XP beads (Beckman Coulter Genomics, Brea, CA, USA) following the manufacturer’s instructions.

### Genome assembly

Raw sequencing data was generated using Illumina base-calling software CASAVA v1.8.2 (http://support.illumina.com/sequencing/sequencing_software/casava.ilmn) according to the manufacturer’s instructions. Contaminated reads, such as those containing adaptors or primers were identified using Trimmomatic (http://www.usadellab.org/cms/uploads/supplementary/Trimmomatic) with default parameters. The clean data obtained were used for further analysis. The *L. salivarius* genome was sequenced using a combination of PacBio RS and Illumina sequencing platforms. The Illumina data were used to evaluate the complexity of the genome and correct the PacBio long reads. First, we used ABySS (http://www.bcgsc.ca/platform/bioinfo/software/abyss) to perform genome assembly with multiple-kmer parameters and obtained the optimal results [19]. Second, canu (https://github.com/marbl/canu) was used to assemble the PacBio corrected long reads. Finally, GapCloser software was subsequently used to fill up the remaining local inner gaps and correct the single base polymorphism (https://sourceforge.net/projects/soapdenovo2 /files/GapCloser/) for the final assembly results [20]. The sequencing raw data have been deposited into the Sequence Read Archive Database of NCBI; the BioProject accession number is PRJNA601071, and the BioSample accession number is SAMN13841246.

### Genome annotation

For the prokaryotic organisms, an ab initio prediction method was used to obtain gene models for strain xx. Gene models were identified using Glimmer3 [21]. All gene models were subjected to BLAST against the non-redundant nucleic acid (NA in NCBI) database, SwissProt (http://uniprot.org), Kyoto Encyclopedia of Genes and Genomes (KEGG, http://www.genome.jp/kegg/) [22], and Clusters of Orthologous Genes (COG, http://www.ncbi.nlm.nih.gov/COG) [23] to perform functional annotation using BLASTP module. In addition, transfer (t)RNAs were identified using the tRNAscan-SE (v1.23, http://lowelab.ucsc. edu/tRNAscan-SE) [24] and rRNAs [25] were determined using the RNAmmer (v1.2, http://www.cbs.dtu.dk/services/RNAmmer/).

### Non-targeted metabonomic analysis

*L. salivarius* was cultured in MRS medium for 16 h; the cultured liquid was filtered at 0.45 μm to harvest the bacteria-free fermented liquid. Then, 100 μL MRS medium or samples of *L. salivarius* fermentation liquid were mixed with 800 μL extract liquid composed of methanol and acetonitrile (volume ratio of 1:1) containing 0.02 mg/mL 2-chlorophenylalanine as an interior label, vortexed for 30 s, and underwent low-temperature ultrasonic extraction (5 ℃, 40 kHz). The samples were refrigerated at − 20 ℃ for 30 min and centrifuged at 13,000x*g* for 30 min. Subsequently, the supernatant was reconstituted with 120 µL water resolution containing 50% acetonitrile and transferred into a vial for ultra-performance liquid chromatography-tandem mass spectrometry (UPLC-MS) analysis [26]. Twenty microliters of each sample were mixed into a quality control (QC) sample for error correction. An ACQUITY UPLC HSS T3 column (2.1 × 100 mm, 1.8 mm; Waters, MA, USA) was used at 40 ℃ column temperature with an injection volume of 2 µL in both positive and negative ion modes. The mobile phase consisted of 0.1% formic acid (Merck, Germany) in water as solvent A and 0.1% formic acid in acetonitrile (Merck, Germany) as solvent B. The flow rate of the mobile phase was 0.25 mL/min. The mass spectrometric parameters were as follows: Electrospray ionized-ion source (ESI), positive ion spray voltage was 3.50 kV, negative ion spray voltage was − 3.50 kV, sheath gas flow rate 50 arb, and auxiliary gas 13 arb. The capillary temperature was 325 ℃, the full scan was performed with a resolution of 60,000, the scanning range was 7,500, the normalized collision energy was 40 eV, and dynamic elimination was used to remove unnecessary MS/MS information [27]. Substances corresponding to the selected metabolites were obtained by searching the freely accessible KEGG databases [28].

### Biochemical assays for digestive enzymes secretion and antimicrobial activity

*L. Salivarius* was cultured in MRS medium for 16 h at 37 ℃, and the fermented liquid was harvested to measure the activity of the digestive enzyme and antimicrobial activity. The activities of protease, α-amylase, β-amylase, and lipase were analyzed using biochemical assays as previously described [29]. The kits were purchased from Nanjing Jian Cheng Bioengineering Institute (Nanjing, China). The assay of antimicrobial activity of *L. salivarius* was analyzed using the cylinder-plate method as previously described [30]. Pathogenic bacterial strains of *Escherichia coli* CCTCC25922, *Salmonella* CVCC1880, and *Staphylococcus aureus* CVCC1882 were purchased from the China Veterinary Microbiology Preservation and Management Center. The bioassay plates were incubated at 37 ℃ aerobically for 18 h. Zone sizes (in millimeters) were carefully measured. Each assay was performed in triplicate, to correct for intra-assay variations. Data were analyzed using SPSS 20.0 (SPSS, Inc., Chicago, IL, USA) and are presented as the mean ± standard error (SE).

## Results

### Phylogenetic analysis and genetic profile

**Fig. 1:**
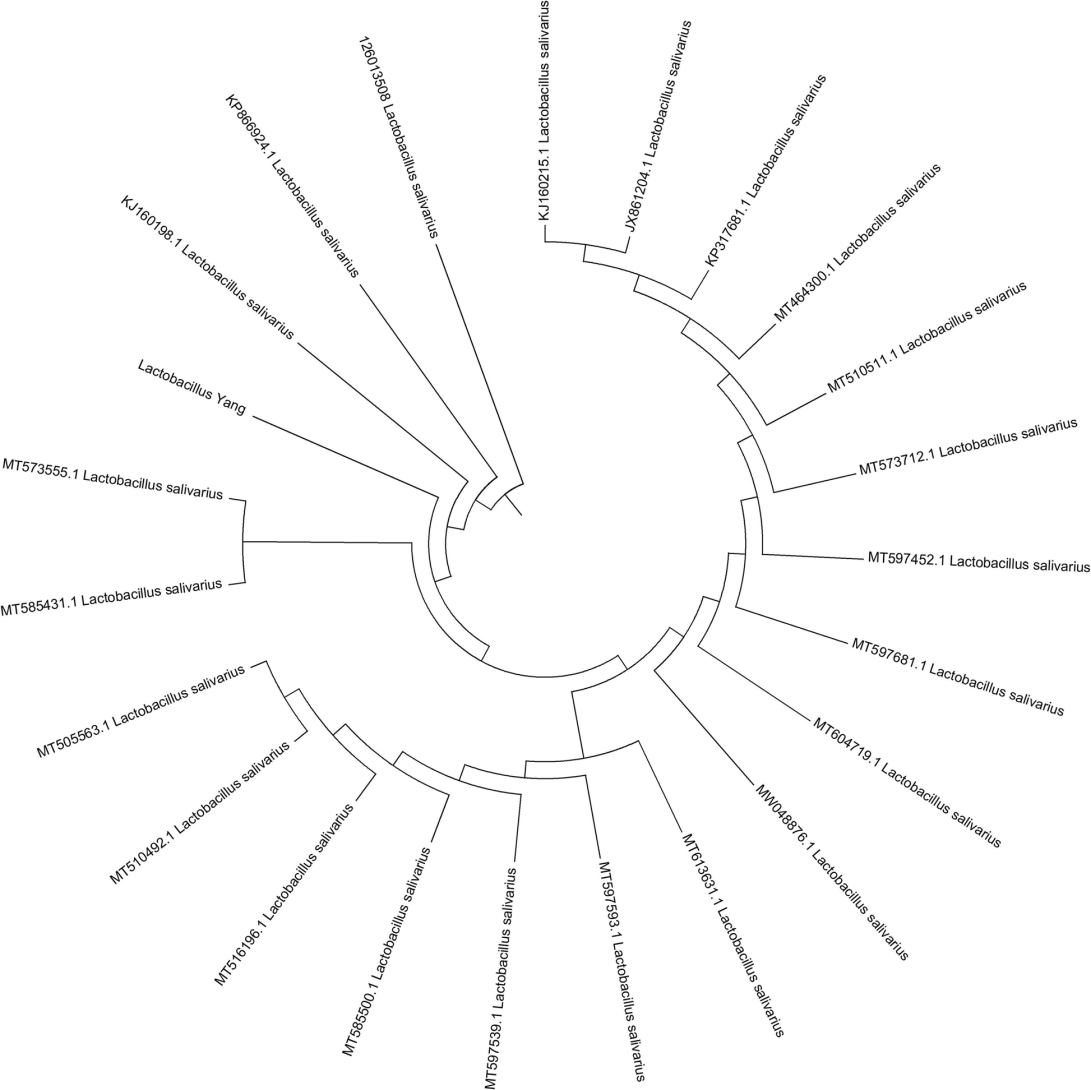
The phylogenetic tree of *Ligilactobacillus salivarius*. Phylogenetic tree of *Lactobacillus salivarius* was drawn employed with MEGA 4.1 software. 16S ribosomal deoxyribonucleic acid (rDNA) of *Lactobacillus salivarius* was extracted to sequence and blast the phylogeny, compared with 22 strains from different origin. *Lactobacillus salivarius* (with green square marked) was closer with *Lactobacillus salivarius MT57355.1* and *Lactobacillus salivarius MT585431.1*. The sequence of *Lactobacillus salivarius* was submitted to NCBI and acquired the ID number of *Lactobacillus salivarius MH517354*. The total number of base pairs (bps) of 16S rDNA was 1542

**Fig. 2:**
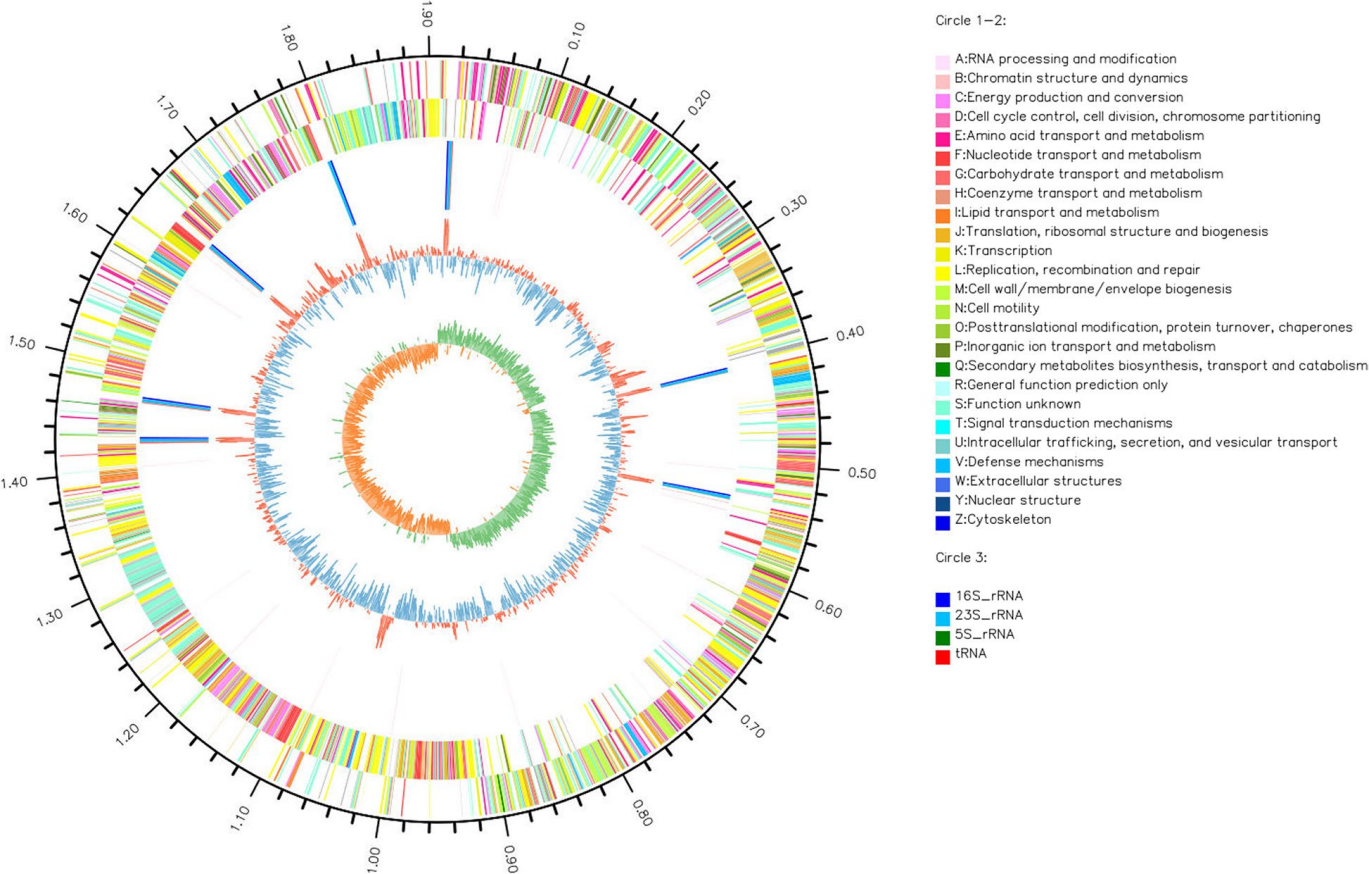
Map of *Ligilactobacillus salivarius* genome. The analysis was performed with Circos v0.64 software (http://circos.ca/). The first circle was the mark of genome size. The second and third circles were functional classification on the coding DNA sequence (CDS) in positive-and negative-chains, using the annotation of Clusters of Orthologous Group (COG). The fourth circle was the ribosomal RNA (rRNA) and transport RNA (tRNA). The fifth cycle was the content of nucleic acid base G and C. The higher the peak value meant the greater the content of G and C, towards inner blue part and the red part towards outer space indicates that the content of G and C was lower and higher than average level. The six circle was the value of GC skew, which was calculated G-C/G + C. The inner green part meant the value was positive, which the content of G was higher than C, represented the positive chain more tendency to transport the CDS. The inner brown part meant the value was negative, which the content of G was negative than C, represented the negative chain more tendency to transport the CDS

DNA was extracted from the precipitate of the cultured broth of *L. Salivarius*. The sequences of 16S rDNA, partial sequences were submitted to NCBI and acquired accession No. MH517354.1. The total number of base pairs (bps) of 16S rDNA was 1542. Comparing it with the 22 strains of *L. salivarius* from the NCBI gene bank, *L. salivarius* MH517354 was closer to *L. salivarius* MT57355.1 and MT585431.1 (Fig. 1). The entire genome of *L. salivarius* was sequenced, the total length of which was 2,255,920 bp; one chromosome and three plasmids genomic were identified, and the G and C contents were 32.9%. Hundreds of functional genes were annotated, including transporters, membrane proteins, some enzymes, heavy metal resistance, and putative protein genes (Fig. 2).

**Fig. 3:**
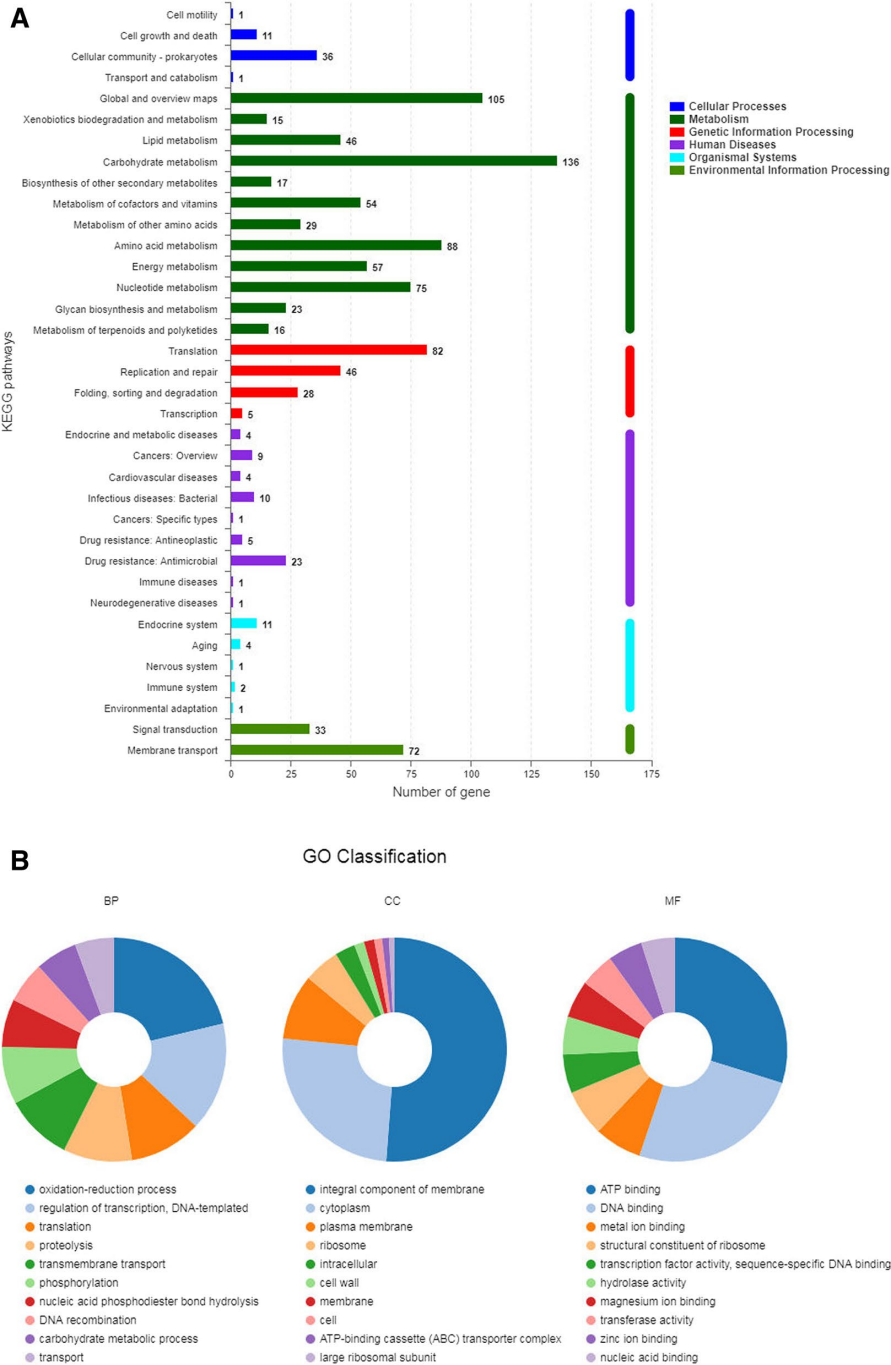
Functional genetic and metabolic profiles on *Ligilactobacillus salivarius.* **A** Prediction of functional genes. Prediction of functional genes through KEGG (Kyoto Encyclopedia of Genes and Genomes, KEGG, http://www.genome.jp/kegg/) pathway. The name of functional genes and the related pathway can be captured for further annotated in total. The number of gene on metabolism of *L. salivarius* was most abundant. **B** Annotation of carbohydrate metabolic gene of *Ligilactobacillus salivarius*. Classification of gene ontology (Go classification) on *Ligilactobacillus salivarius.* Functional genes on genetic biological process (BP), cellular component (CC) and molecular function (MF). The pie graphics showed the top ten number of genes on the proportion of BP, CC, and MF. Among BP genes, the highest number was the oxidation–reduction process, to decline the antioxidation levels in environment. Among CC, most of number of genes were related with plasm of bacterial cell. In MF genes, the binding on adenosine triphosphate (ATP), deoxyribonucleic acid (DNA) and metal ion was covered three top proportions

The genes of *L. salivarius* coding proteins were predicted (Fig. 3A). The number of genes involved in metabolism was the highest at 661, while 161 genes were associated with genetic information processing, comprising the second position in the total ratio. The number of genes involved in environmental information processing, human diseases, cellular processes, and organismal systems were 105, 58, 49, and 19, respectively. To further distinguish the functional genes involved in genetic biological process (BP), cellular component (CC), and molecular function (MF), the genetic and metabolic information analysis of gene ontology (GO) was performed; the top 10 genes for each are shown in Fig. 3B. Among the BP genes, the highest number was associated with the oxidation–reduction process, which reduces environmental antioxidation levels. In CC, most genes were associated with the plasma membrane of bacterial cells. Among the MF genes, the binding of adenosine triphosphate (ATP), DNA, and metal ion covered the top three proportions.

### Genetic prediction of *Lactobacillus salivarius* for virulence factors

**Fig. 4:**
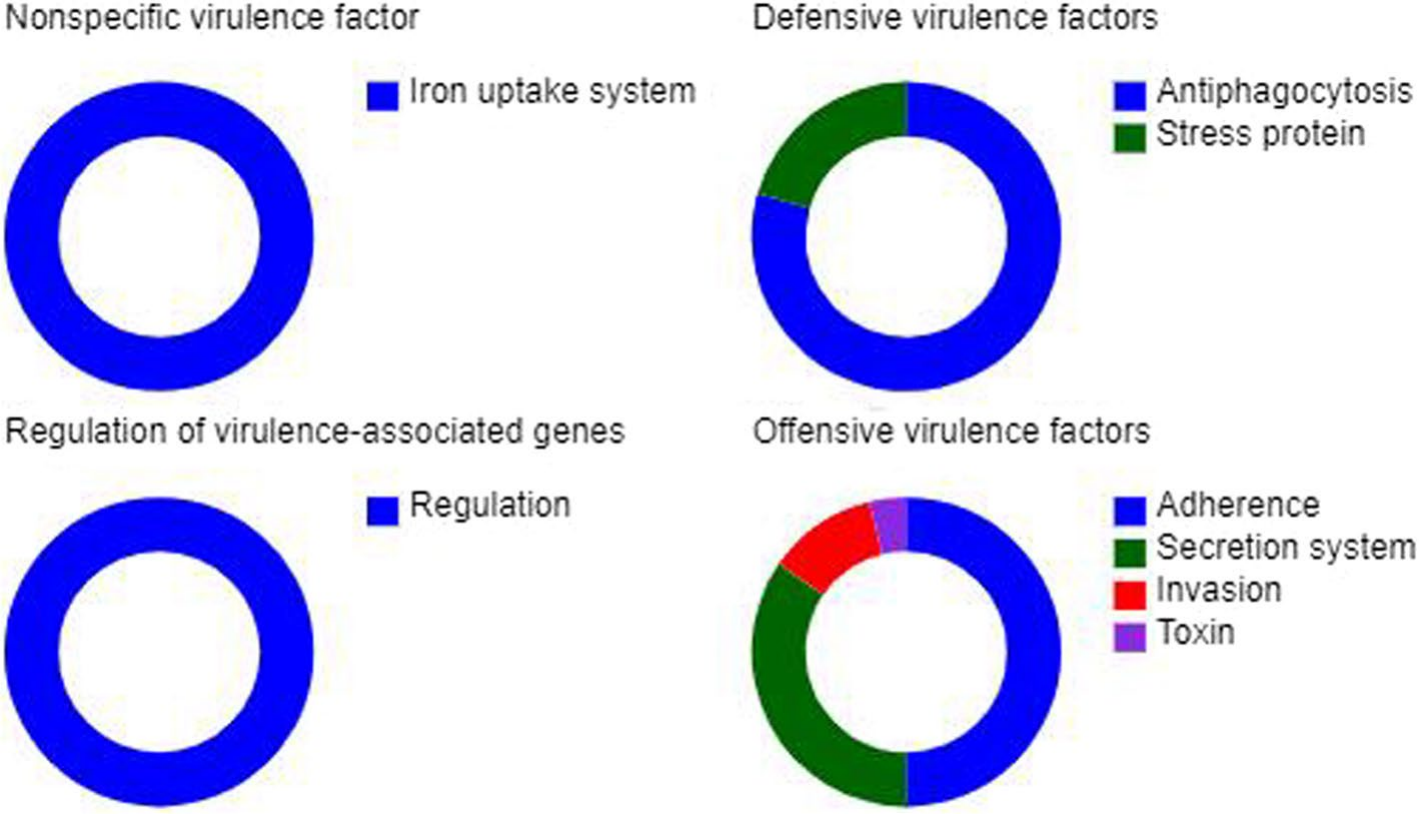
Prediction of *Ligilactobacillus salivarius* for virulence factors and environmental adaptation. The classification of genetic prediction for virulence factors. When *Ligilactobacillus salivarius* was colonized in host, the possible of risk were evaluated through prediction of genetic information. The virulent factor statistic on *Ligilactobacillus salivarius* manifested that there were nonspecific virulence factors except the iron uptake system, which is fitful to bodily iron absorption. Also, the regulation of virulence associated gens were none in cells of *Ligilactobacillus salivarius.* In order to defense the cell, two protected gene namely antiphagocytosis and stress protein were contained. The prediction of gene showed that the adherence of *Ligilactobacillus salivarius* owned when administrated and entered gastrointestinal tract and adopt to the physiological conditions so that colonized in mucous of intestine and propagated continuously

The prediction results of the evaluation of the virulence of *L. salivarius* in the host are shown in Fig. 4. The virulence factor statistics manifested were non-virulence factors, containing an iron uptake system beneficial for bodily iron absorption. Additionally, the regulation of virulence-associated genes was not observed. To protect cells from stress, two protective genes anti-phagocytosis and stress protein were identified. Genetic predictions showed that *L. salivarius* possess the capacity for adherence to biofilms. In the annotation of genetic offensive virulence factors, the capacity of adherence and secretion system account for the highest ratio with invasion and toxin occupying a small proportion in total. There were six types of secreted systems (Supplementary Fig. 1). The substances secreted were mainly composed of proteins and peptides. Secretory proteins ere synthesized by *L. salivarius* and were digestive enzymes, antibodies, hormones, and pre-proteins involved in regulating apoptosis.

### Digestive enzymes related genes and activities

**Fig. 5:**
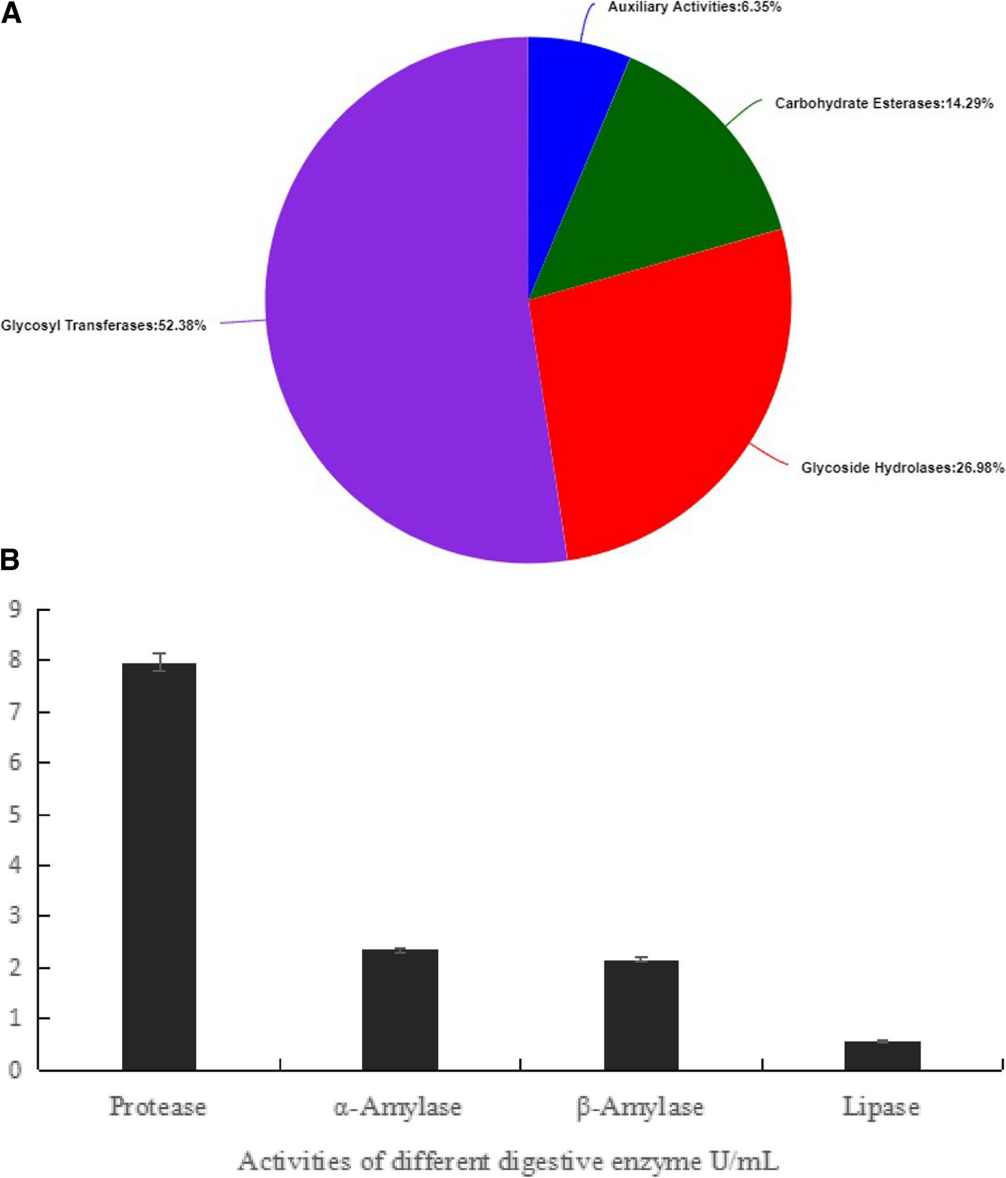
Digestion related genes and their capacity. **A** Annotation of carbohydrate metabolic gene of *Ligilactobacillus salivarius* respectively. Glycosyl transferase covered 52.38% in total proportion. Glycoside hydrolases, carbohydrate esterases, and auxiliary activities covered 26.98%, 14.29% and 6.35% respectively. **B** Capacity of protease, α-amylase, β-amylase, and lipase containing in fermentation of *Ligilactobacillus salivarius*. Live number of *L. salivarius* in per mL reached 1.0 × 10^8^ CFU/mL. The activity of protease, α-amylase, β-amylase, and lipase reached 7.97, 2.34, 2.16, and 0.56 U/mL containing 10^8^ CFU *L. salivarius*

The annotation of the carbohydrate metabolism genes in *L. salivarius* is shown in Fig. 5A. During glycose lysis and digestion, there were genes for glycoside hydrolases, carbohydrate esterases, auxiliary activities, and glycosyl transferases. Glycosyl transferase accounted for 52.38% of the total, glycoside hydrolases covered 26.98% of the total enzymes, and carbohydrate esterases and auxiliary activities accounted for 14.29% and 6.35%, respectively. In nutritional substance digestion, *L. salivarius* catalyzes glycogen, protein, and fatty acid digestion, thereby facilitating absorption of these molecular substances by the host. The activities of protease, α-amylase, β-amylase, and lipase in the fermentation of *L. salivarius* are shown in Fig. 5B.

The number of live *L. salivarius* per milliliter of fermentation liquid reached 1.0 × 10^8^ colony forming units per milliliter (CFU/mL). The activities of protease, α-amylase, β-amylase, and lipase were 7.97, 2.34, 2.16, and 0.56 U/mL, respectively.

### Functional metabolite in fermentation of *L. salivarius MH517354*

Functional genetic perdition revealed that metabolism-related genes were the most abundant. The metabolism of phenylalanine in *L. salivarius* was more significant in the fermentation medium than in the control MRS medium (*P* < 0.001), as shown in Supplementary Fig. 2A. The metabolism of arginine and d-ornithine was significantly higher (*P* < 0.01) when the bacteria colonized the MRS medium. The purine, glutathione, and alpha-linolenic acid content and secondary metabolite biosynthesis in fermented medium significantly increased (*P* < 0.05) with the growth of *L. salivarius*. The concentrations of ceramide, Pyrrolidone-5-carboxylic acid, N2-Acetyl-L-ornithine, 2-Ethyl-2-Hydroxybutyric acid, 2-Ethyl-2-Hydroxybutyric acid, N-lactoyl-phenylalanine, and 14 other substances contained in the fermentation of *L. salivarius MH517354* were significantly higher than the control of MRS cultural medium (*P* < 0.001; Supplementary Fig. 2B). Metabolites produced by *L. salivarius*, such as ceramide and phenylalanine, are functional substances in the body.

### Antimicrobial activities and genetic prediction

**Fig. 6:**
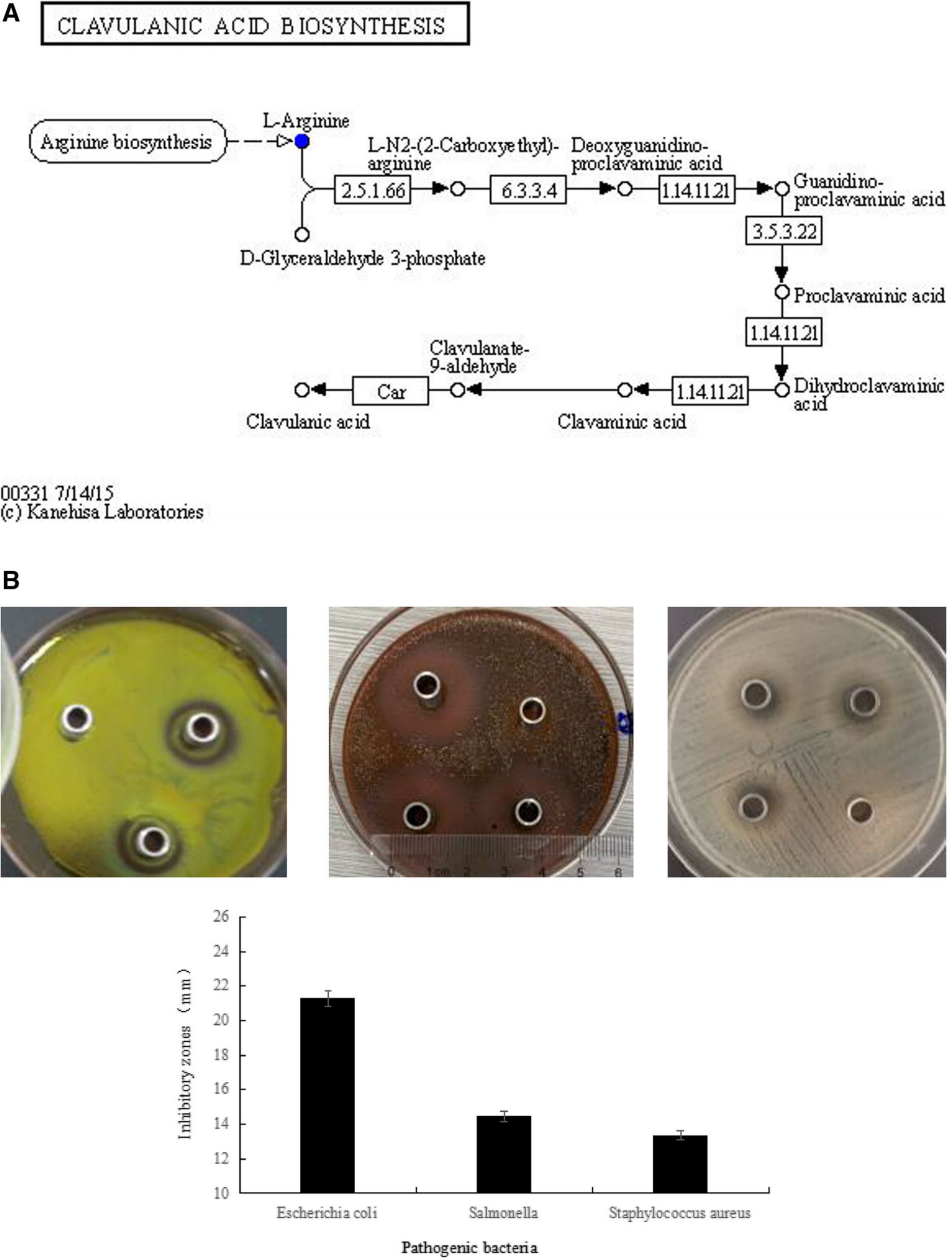
The profile of antimicrobial activity on *Ligilactobacillus salivarius*. **A** The inhibitory action of *Ligilactobacillus salivarius* on pathogen and the metabolic profiles. The biosynthesis pathway was annotated depending on KEGG. The graph of metabolic pathway was found through enriched of KEGG metabolic pathway after collecting the metabolites through UPLC-MS detection. Clavulanic acid produced by *L. salivarius* and their pathway were manifested comprehensively. The pathway was originated from the metabolism of arginine through the cascade catalyzed reactions, the processed substances were L-N2-(2-Carboxye thyl)-arginie, Deoxyguanidion-proclavaminic acid, Guanidino- proclavaminic acid, Proclavaminic acid, Dihydroclavaminic acid, Clavaminic acid, Clavulanate-9-aldehyde in sequenced. Clavalanic acid was produced in the end. **B** The inhibitory action of *Ligilactobacillus salivarius* on pathogen. The inhibitory action of *Lactobacillus salivarius* on pathogen. The classic cylinder-plate method was employed to evaluate the antimicrobial activity. Three strains of common pathogenic bacteria: *Escherichia coli*, *Salmonella*, *Staphylococcus aureus* were used as the test object. The inhibitory zones were measured to evaluate the antimicrobial capacity

The bacteriocins produced by *L. salivarius* were analyzed after metabolite collection using UPLC-MS; a distinct analysis of the metabolic substances is shown in Supplementary Fig. 2B. Five types of antimicrobial metabolites were secreted, namely phenyllactic acid, Janthitrem G, 13-demethyl tacrolimus, Medinoside E, and Tertonasin compared with the control MRS medium (*P* < 0.001). The enriched functional substances were analyzed using KEGG, and the biosynthesis of one antibiotic clavulanic acid was clarified. The biosynthesis pathway is presented in Fig. 6a. The pathway originated from the metabolism of arginine through the cascade catalyzed reactions, and the processed substances were L-N2-(2-Carboxye thyl)-arginie, Deoxyguanidion-proclavaminic acid, Guanidino- proclavaminic acid, Proclavaminic acid, Dihydroclavaminic acid, Clavaminic acid, and Clavulanate-9-aldehyde in sequenced. Clavalanic acid was produced at the end.

The metabolites were compared with those in the human metabolome database. One bacteriocin synthesized by *L. salivarius* was present in fermentation. Library ID: HMDB0033335, Common name: Antibiotic X 14889C, monoisotopic mass: 614.402998076, superclass: lipids and lipid-like molecules, class: prenol lipids, subclass: sesterterpenoids. The total concentration reached 5.93 µg/L in liquid MRS medium, which was higher by 1.42 µg/L than the control MRS medium.

The antibacterial activity of *L. salivarius* against pathogenic bacteria was evaluated. An inhibitory assay was performed on the three strains of pathogenic bacteria (Fig. 6b). The inhibitory zones of *L. salivarius* on *Escherichia coli* CCTCC25922, *Salmonella* CVCC1880, and *Staphylococcus aureus* CVCC1882 reached 22.37 mm, 19.69 mm, and 20.49 mm, respectively.

## Discussion

We have previously reported that *L. salivarius* improved the growth and health of weanling piglets [7]. Genomic evaluation was first used to obtain the genetic information of *L. salivarius* [18, 31]. The phylogenetic tree suggested that *L. salivarius* was closely related to *Ligilactobacillus salivarius* MT57355.1 and MT585431.1, probiotic bacteria isolated from animals. In addition, the bacteria possessed special characteristics in their genetic composition, GC content, and functions. Annotation of genetic information indicated that the metabolism accounted for most of the genes in *L. salivarius*, and the metabolic genes were focused on carbohydrates, amino acids, nucleotides, and energy. The biosynthesis of secondary metabolites is especially important for the host in the gastrointestinal tract. In addition, environmental information processing and human diseases are potential threats to the host. Hence, GO classification and prediction of virulence factors and environmental adaptations were evaluated. GO classification showed no genes predicted as host threats. Most of the genetic information of *L. salivarius* was associated with its oxidation reduction process, which is beneficial to the host.

The lack of a specific threat was confirmed by the genetic prediction of virulence factors. Considering the aspects of BP, CC, and MF, *L. salivarius* is highly suitable for clinical use.

The analysis of the virulence factors suggested that after supplementation of animals with *L. salivarius*, the bacteria adhered to the intestinal membrane of the mucosa. To survive in the environment, *L. salivarius* must metabolize and secrete digestive enzymes to acquire nutritional substances [7, 10]. The carbohydrate metabolic genes were associated with glycoside hydrolases, carbohydrate esterases, and auxiliary activities. Whether other metabolites secreted by *L. salivarius* play negative effects on bodily health remains to be determined. Six types of secreted systems of the bacteria were clarified, suggesting mainly transmembrane proteins were secreted and signal peptides were hydrolyzed to function as digestive enzymes. The activity of protease, α-amylase, β-amylase, and lipase, which are involved in digestion [3], secreted in the fermentation medium by *L. salivarius* was measured [3]. The ability to adhere enhances probiotic effects, which use the nutrition in the intestine to colonize and metabolize more digestive enzymes to play reciprocal roles in the host [11].

Non-targeted metabonomic techniques are often employed to explore the unknown metabolites produced by bacteria [32, 33]. To further reveal the metabolic differences, KEGG and distinct analyses were performed to determine the beneficial molecules and their concentrations based on metabonomic assays. Besides the digestive enzyme and adhered protein secreted, our results suggested that metabolism and biosynthesis of amino acids, especially arginine, proline, and phenylalanine, were significantly regulated by enzymes secreted by *L. salivarius*, which improved the concentration of nitrogenous compounds such as ceramide, pyrrolidone-5-carboxylic acid, N2-acetyl-L-ornithine, and 2-ethyl-2-hydroxybutyric acid. Ceramide, pyrrolidone-5-carboxylic acid, and N2-acetyl-L-ornithine were involved in antioxidation and cellular membrane repair to protect the cuticle of the skin against exogenous stimuli [34–36]. 2-ethyl-2-hydroxybutyric acid is an anticonvulsant and hypnotic agent that can improve sleep [37, 38]. N-lactoyl-phenylalanine, secreted after physical exercise, inhibits appetite in obese individuals [39, 40].

Antimicrobial metabolites produced by *L*. *salivarius* were also annotated. One macromolecular substance, clavalanic acid, which has antimicrobial properties, produced by *L. salivarius* was annotated via the KEGG enriched pathway [41]. The pathway stems from the metabolism of arginine through the cascade catalyzed reactions; the processed substances were L-N2-(2-Carboxye thyl)-arginine, deoxyguanidion-proclavaminic acid, Guanidino- proclavaminic acid, proclavaminic acid, dihydroclavaminic acid, clavaminic acid, and clavulanate-9-aldehyde; clavalanic acid was produced at the end. Clavalanic acid, a β-lactamase inhibitor used as an antibiotic drug against gram-positive bacteria, is synthesized by *L. salivarius* through cascade-catalyzed reactions. The antibiotic X 14889C is another bacteriocin-synthesized molecule, which has a molecular structure similar to that of streptomycin, is active against gram-positive bacteria, and exhibits ionophore properties [42]. Streptomycin exerts inhibitory effects on gram-negative bacteria through inhibiting the synthesis of bacterial protein. The secretion of other bacteriocins namely phenyllactic acid, Janthitrem G, 13-demethyl tacrolimus, medinoside E, and tertonasin showed antimicrobial activity against pathogenic *Escherichia coli* CCTCC25922, *Salmonella* CVCC1880, and *Staphylococcus aureus* [43–45].

The bacterial metabolites evaluated using the metabonomics assay did not fulfill the predictions made by genomics. The types of antibiotic drugs predicted for *L. salivarius* were more than the metabolites measured using UPLC-MS.

In conclusion, in this study, genomic, metagenomic, and biochemical assays on *L. salivarius* uncovered its beneficial characteristics, in line with a previous animal study, where weanling piglets, supplemented with *L. salivarius* showed colonization of the cecal mucous membrane and optimization of the bacterial composition in the intestine. Our research provides novel insights into the mechanism underlying the interplay between probiotics and hosts, and a database to compare the beneficial characteristics with other probiotics.

### Supplementary Information

**Additional file 1.**

**Additional file 2.**

**Additional file 3.**

**Additional file 4. **Supplementary data-16S genetic sequence.

## Data Availability

The sequencing raw data have been deposited into the Sequence Read Archive Database of National Center for Biotechnology Information (NCBI). The BioProject accession number is PRJNA601071. The accession numbers bioSamples was SAMN13841246.
