# Supplementary figures and images for: Genomic and metabonomic methods reveal the probiotic functions of swine-derived Ligilactobacillus salivarius
Source: BMC Microbiol. 2023 Aug 30;23:242. doi: 10.1186/s12866-023-02993-9 (PMC10466738; doi:10.1186/s12866-023-02993-9)

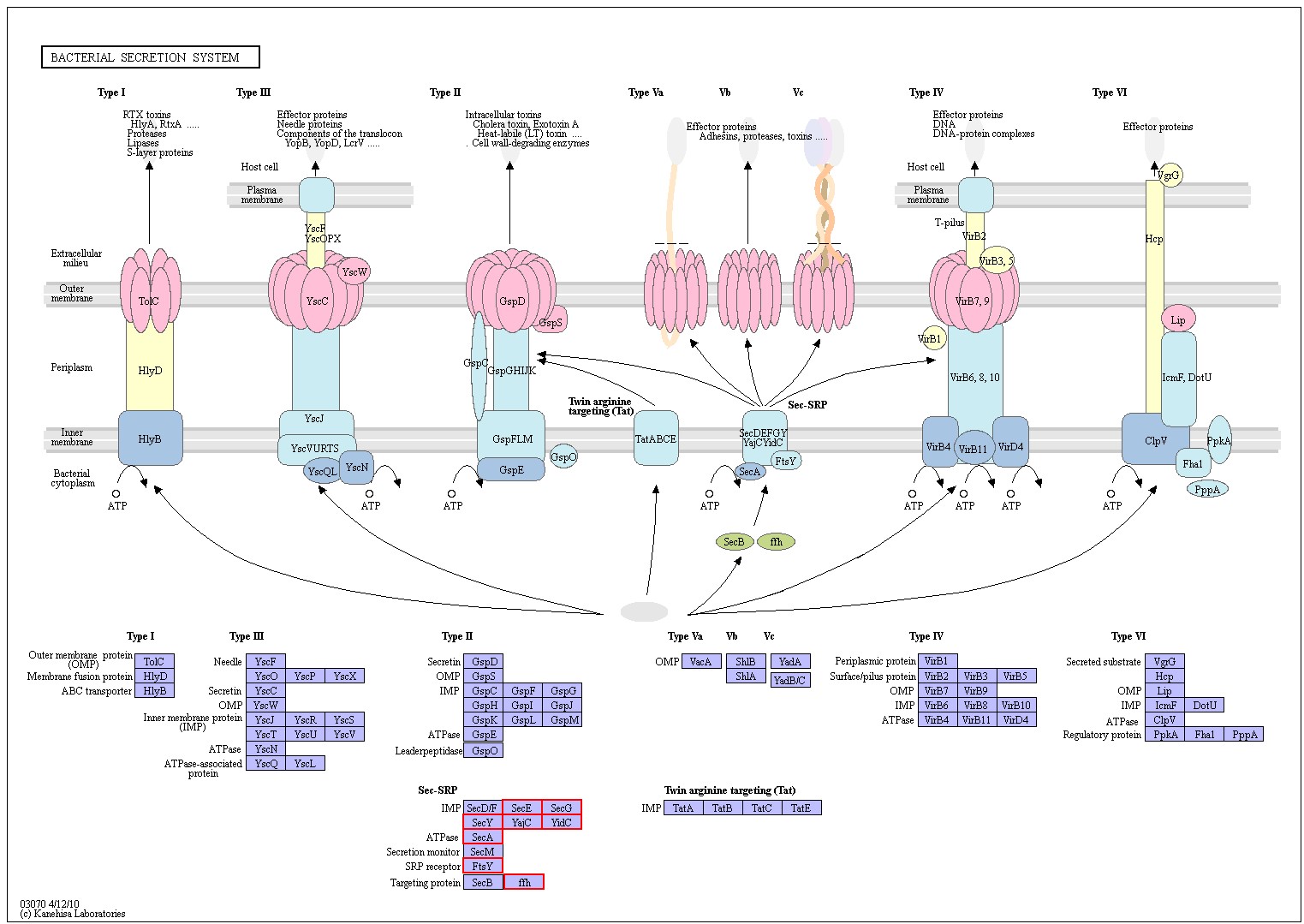

Supplement: Supplementary file 1 — Additional file 1. [file 12866_2023_2993_MOESM1_ESM.jpeg]

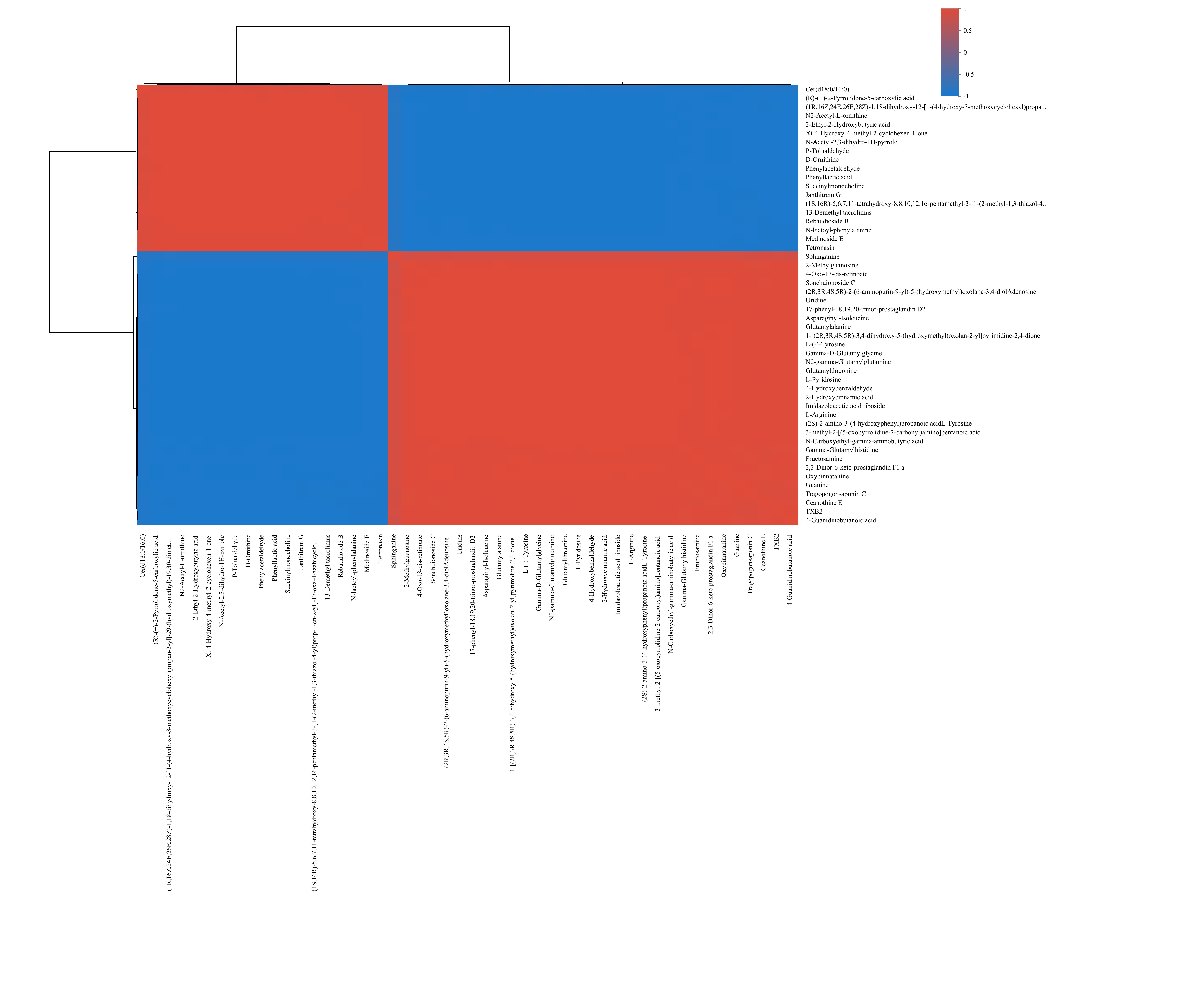

Supplement: Supplementary file 2 — Additional file 2. [file 12866_2023_2993_MOESM2_ESM.jpeg]

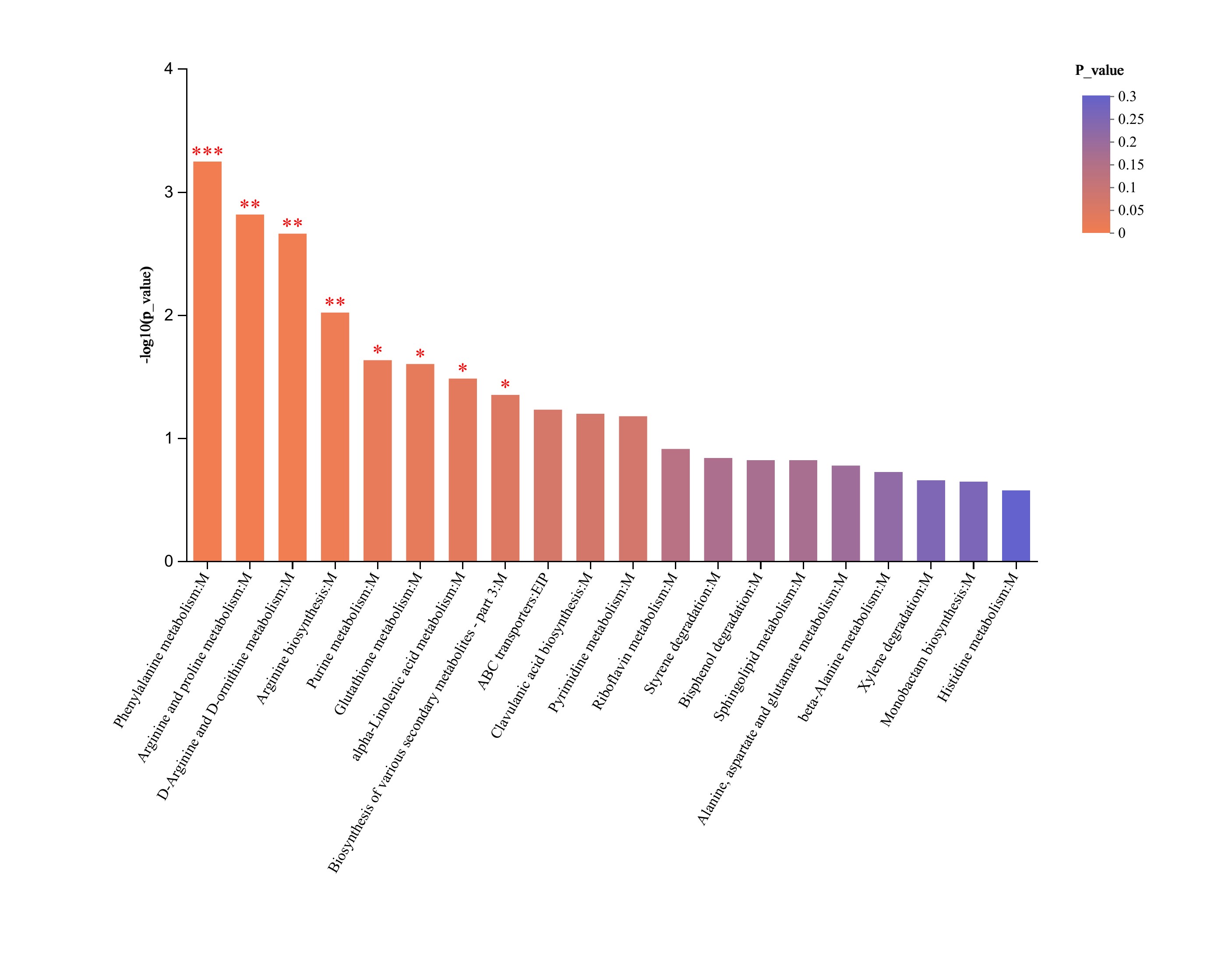

Supplement: Supplementary file 3 — Additional file 3. [file 12866_2023_2993_MOESM3_ESM.jpeg]
